# The media composition as a crucial element in high-throughput metabolic network reconstruction

**DOI:** 10.1098/rsfs.2022.0070

**Published:** 2023-02-10

**Authors:** Benedict Borer, Stefanía Magnúsdóttir

**Affiliations:** ^1^ Earth, Atmospheric and Planetary Sciences Department, Massachusetts Institute of Technology, Cambridge, MA 02139, USA; ^2^ Department of Environmental Microbiology, Helmholtz Centre for Environmental Research-UFZ, Leipzig 04318, Germany

**Keywords:** metagenome-assembled genomes, genome-scale metabolic networks, automated gap filling, KBase, high-throughput reconstruction, amino acid auxotrophies

## Abstract

In recent years, metagenome-assembled genomes (MAGs) have provided glimpses into the intra- and interspecies genetic diversity and interactions that form the bases of complex microbial communities. High-throughput reconstruction of genome-scale metabolic networks (GEMs) from MAGs is a promising avenue to disentangle the myriad trophic interactions stabilizing these communities. However, high-throughput reconstruction of GEMs relies on accurate gap filling of metabolic pathways using automated algorithms. Here, we systematically explore how the composition of the media (specification of the available nutrients and metabolites) during gap filling influences the resulting GEMs concerning predicted auxotrophies for fully sequenced model organisms and environmental isolates. We expand this analysis by using 106 MAGs from the same species with differing quality. We find that although the completeness of MAGs influences the fraction of gap-filled reactions, the composition of the media plays the dominant role in the accurate prediction of auxotrophies that form the basis of myriad community interactions. We propose that constraining the media composition for gap filling through both experimental approaches and computational approaches will increase the reliability of high-throughput reconstruction of genome-scale metabolic models from MAGs and paves the way for culture independent prediction of trophic interactions in complex microbial communities.

## 1. Background

Microbes are the most diverse form of life on our planet [1–3], drive global biogeochemical cycles [4,5] and are vital for human health [6–8]. Despite our best efforts, the vast majority of microbial species or operational taxonomic units (OTUs) remain uncultured [9–12]. Metagenomic analysis and especially their translation into metagenome-assembled genomes (MAGs) have vastly expanded our knowledge of the tree of life [13] and increased the number of available genomes of so-far uncultured species/OTUs across biomes [14]. Although experimental technologies are emerging that permit direct genome editing of individual species in microbial communities *in situ* [15], disentangling multi-faceted species interactions within complex and uncultured communities is confined to the computational realm since isolation may rapidly result in divergent behaviour when compared to their ‘real world behaviour’ [16]. Translating metagenomic data into mathematical frameworks such as genome-scale metabolic networks (GEMs) via MAGs enables culture-independent analyses that uncovered metabolically mediated co-occurrence patterns [17], identified costless metabolic secretions as drivers of interspecies interactions [18], revealed cross-kingdom symbiotic relationships between yeast and lactic acid bacteria via nitrogen overflow [19] or permitted genome-informed isolation of so far uncultured species [20], among myriad other applications. GEMs are traditionally reconstructed from complete genome sequences and manually curated to reconcile model predictions with experimental observations [21]. In recent years, multiple semi-automated algorithms have emerged that enable high-throughput reconstruction of GEMs from genome sequences [22–25], and further developments of computational techniques [26] and pipelines [27] are promising endeavours towards disentangling complex interactions in uncultured microbial communities. However, our inability to compare the reconstructed GEMs to experimental data requires confidence in the reconstruction process. There are multiple processes that contribute to uncertainty during metabolic network reconstruction such as the functional annotation of genes encoding metabolic enzymes (gene–protein–reaction association), the definition of the media used for gap filling, the algorithm used for gap filling of the draft metabolic network or the formulation of the species-specific biomass composition, which have been recently reviewed [28]. When reconstructing GEMs from MAGs to assess culture independent community interaction patterns, precise gap filling of metabolic pathways is crucial to predict potential auxotrophies (i.e. the inability of an organism to synthesize a biomass precursor required for its growth). Auxotrophies form the basis of many metabolic interactions [29–31] and may fundamentally alter the interactions between species in complex communities [32]. Auxotrophies are ubiquitous in the microbial world, and comparative genomics has revealed that the majority of microbial species cannot synthesize all 20 amino acids and thus rely on community interactions to proliferate [29,33]. To accurately predict metabolic interactions in complex communities from genome sequencing alone, we need to better understand how the media composition for gap filling influences the resulting metabolic network.

Here, we use bacterial genomes ranging from isolated model species to MAGs of varying quality to assess the importance of the media composition during automated metabolic network reconstruction. We employ KBase, the US Department of Energy Systems Biology Knowledgebase [34] for the annotation, reconstruction and gap filling of the metabolic networks. We chose KBase as it is a publicly available and user-friendly platform that enables rapid reconstruction of GEMs from sequences without substantial bioinformatic knowledge requirements. We analyse all resulting networks by observing predicted auxotrophies when growing in media that differ in their composition when compared to the media composition used for reconstruction. We focus on predicted auxotrophies for all species as a function of imposed media composition during gap filling as these often form the basis of trophic interactions in complex bacterial communities. Gaining a better understanding of how the potential bias introduced during the reconstruction procedure shapes the prediction from community models will be vital in future studies where metagenomic data are translated to functional metabolic community models to access and predict interactions within the uncultured ‘dark microbial biomass’.

## 2. Results and discussion

### 2.1. Media composition during reconstruction determines auxotrophies of resulting metabolic networks

**Figure 1.**
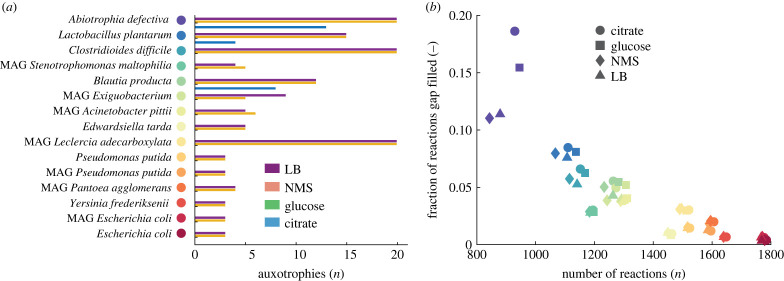
Predicted auxotrophies and relation to gap-filled reactions. (*a*) Number of predicted auxotrophies for growth in glucose minimal media when reconstructed using different media compositions for automated gap filling. (*b*) Percentage of the reactions in a network was gap filled in relation to the network size. The colour scheme for the species is shown in (*a*). LB: rich complex medium, NMS: defined rich medium, glucose: defined minimal media with glucose as carbon source, citrate: defined minimal media with citrate as carbon source.

To observe the potential bias concerning auxotrophies introduced during automated gap filling, we reconstructed a total of 15 GEMs that include eight whole-genome sequences of isolated species (*Escherichia coli* K12 substr. MG1655, *Pseudomonas putida* B6-2, *Yersinia frederiksenii* ATCC 33641, *Lactobacillus plantarum* JDM1, *Edwardsiella tarda* ATCC 23685, *Clostridioides difficile* CD196, *Blautia producta* ATCC 27340 DSM 2950 and *Abiotrophia defectiva* ATCC 49176) and seven sequences from MAGs (*Escherichia coli, Pseudomonas putida*, *Pantoea agglomerans*, *Leclercia adecarboxylata*, *Exiguobacterium* ssp. and *Acinetobacter pittii*). All seven MAGs were of high quality with a completeness greater than 90% and contamination less than 5%. For each strain, we reannotated the genome sequence using RASTtk v1.073 [35] and reconstructed the GEM using four different media compositions (two defined minimal media, a defined rich medium and a complex rich medium) for automated gap filling (for details, see methods section). We subsequently quantified auxotrophies for amino acids for all reconstructed GEMs when growing on minimal media supplemented with glucose using the COBRA toolbox (figure 1*a*). Quantification of auxotrophies for all combinations (i.e. media used for gap filling versus simulated media composition for growth) can be found in electronic supplementary material, figure S1. Most networks that were reconstructed with rich media for gap filling (NMS and LB) resulted in numerous amino acid auxotrophies when simulating growth in glucose minimal media (figure 1*a*). In this case, amino acids are abundant and only transport reactions were added during gap filling whereas reactions required for their biosynthesis were omitted. This contrasts with many bacterial species that may have transport reactions for the different amino acids, but also contain the full set of genes for biosynthesis. For instance, the two strains *Escherichia coli* K12 *substr*. MG1655 [36] and *Pseudomonas putida* B6-2 [37] readily grow in defined minimal media in the absence of any amino acids whereas reconstruction using complex media predicts numerous auxotrophies. At the other end of the spectrum, most reconstructions that used a minimal media composition for gap filling did not show any auxotrophies when growing on minimal media. This may result in a reduced number of interdependencies between the species and thus bias the predicted community interaction patterns. For instance, *C. difficile* infections are typically associated with elevated amino acid availability [38] and rely on provision of multiple amino acids from other species in the community for proliferation. Similarly, ‘Abiotrophia’ means life nutrition deficiency and refers to the species’ dependance on available biomass precursors for growth [39]. The root of this bias comes from the gap filling algorithm itself. When minimal media is used for reconstruction and gap filling, all missing reactions that are required to create biomass precursors (that include amino acids) are added during automated gap filling to produce flux through the biomass reaction. In many cases, this results in the addition of myriad reactions to synthesis pathways for biomass precursors and may make up a substantial amount of the total reactions in a metabolic network, especially for smaller genomes (figure 1*b*). For *Abiotrophia defectiva*, automated gap filling contributed up to 18% of all reactions to the metabolic network and thus fundamentally altered the organisms’ metabolic capabilities. Together, these results highlight that defining a realistic nutrient composition for automated gap filling is crucial when reconstructing metabolic networks of uncultured strains or from MAGs where comparison of predicted auxotrophies with experimental data is not possible.

**Figure 2.**
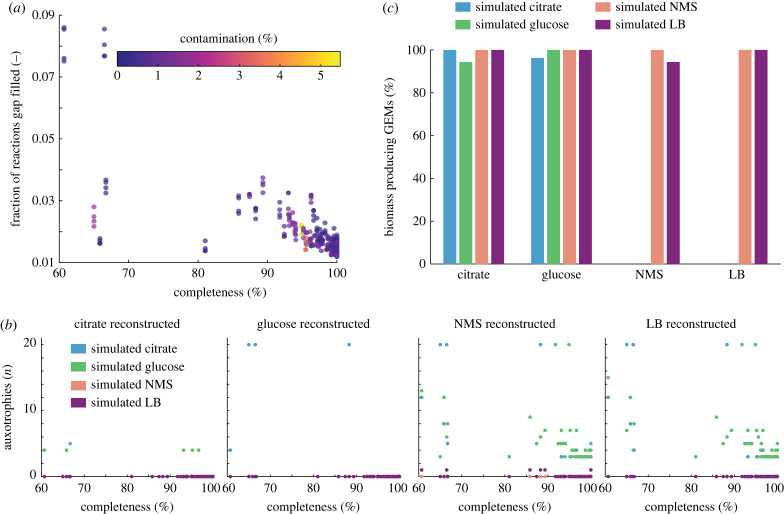
Predicted auxotrophies for 106 GEMs reconstructed from MAGs of the same species with variable completeness and contamination. (*a*) Relation between MAG completeness, contamination and fraction of reactions that were gap filled during reconstruction. (*b*) Number of predicted auxotrophies when simulated in different growth media as a function of the media composition used for reconstruction. (*c*) Percentage of the GEMs reconstructed from MAGs using different media compositions for gap filling (indicated on the *x*-axis) that can produce biomass using the different media compositions for growth (colour scheme).

Recently, pipelines for predicting auxotrophies from genome sequences have been developed [40,41]. AuxoFind [40] uses available GEMs and curated genome sequences to find conditionally essential genes that are required for growth but can be compensated for by external nutrients (i.e. the absence of a conditionally essential gene indicates an auxotrophy). By contrast, GapMind [41] uses a pathway-centric approach and relies on experimentally characterized proteins to reconstruct and annotate biosynthesis pathways of amino acids in prokaryotic genomes and provides confidence estimates (a comparison for auxotrophies predicted by reconstructed GEMs versus GapMind is shown in the electronic supplementary material, figure S2). These algorithms are precious tools for predicting auxotrophies mechanistically but rely on complete genome sequences that may not be available for MAGs depending on their quality. To investigate the influence of MAG completeness and contamination on potential auxotrophies, we reconstructed 106 GEMs from MAGs of the genomic OTU *Pseudomonas_A nitritolerans* ranging from a completeness of 60% to 100% and contamination from 0% to 5.5%. As expected, the fraction of gap-filled reactions in the network increases with a decrease in completeness whereas contamination does not have a specific discernable influence (figure 2*a*). This is primarily driven by the incomplete genome sequence and the need for inferring the missing reactions using automated gap filling. Three bins with low completeness required only marginal gap filling. When contrasting these bins with two of low completeness but substantial gap filling, no evident pattern emerges across the different metabolic pathways (electronic supplementary material, figure S3), and the result is most likely due to randomness in the completeness of the bins.

When reconstructing the GEMs using different media composition for gap filling, no clear pattern emerges between the MAG completeness and the predicted auxotrophies (figure 2*b*, table 1). Overall, no auxotrophies are predicted for GEMs reconstructed using a defined minimal media composition (citrate and glucose minimal media) when simulated growth in rich media (table 1). However, six bins show auxotrophies when simulating growth in glucose minimal media and three bins when simulating growth in citrate minimal media for metabolic networks reconstructed on citrate and glucose media, respectively. By contrast, GEMs that are reconstructed with rich media (NMS and LB) show a high abundance of auxotrophies when simulating growth on defined minimal media (citrate and glucose, table 1). In summary, an average of 4.22 auxotrophies are predicted for metabolic networks reconstructed in rich media and simulated in defined media whereas no auxotrophies are predicted for metabolic networks reconstructed in defined media but simulated in rich media. Interestingly, the quality of the MAGs shows no influence on the average predicted number of auxotrophies when contrasting high quality (greater than or equal to 90% complete) with medium quality (less than 90% complete) MAGs (electronic supplementary material, table S1).

**Table 1.** Average number of auxotrophies for all GEMs derived from MAGs depending on the reconstruction media and simulation media. Each row corresponds to a reconstruction media, the corresponding simulated media are shown as columns. Data are shown as mean with s.d. in brackets.

		**simulation media**			
		citrate	glucose	NMS	LB
reconstruction media	citrate	*0* (*0)*	*0.24* (*0.97)*	*0* (*0)*	*0* (*0)*
	glucose	*0.60* (*3.34)*	*0* (*0)*	*0* (*0)*	*0* (*0)*
	NMS	*4.32* (*3.73)*	*4.08* (*2.79)*	*0* (*0)*	*0.06* (*0.23)*
	LB	*4.36* (*3.73)*	*4.16* (*2.87)*	*0* (*0)*	*0* (*0)*

Congruent to the analysis for whole-genome sequences, this stark difference is due to the media composition used for gap filling since all biosynthetic pathways are gap filled when reconstructed using a defined minimal media whereas only transport reactions are added in the case of rich media. As a result, most GEMs reconstructed with defined media can produce biomass irrespective of the media composition used for simulating growth whereas GEMs reconstructed with rich media for gap filling are unable to produce biomass when simulating growth on defined media (figure 2*c*; electronic supplementary material, table S2).

Evidently, the choice of media composition for gap filling during metabolic network reconstruction is consequential for the resulting network structure, growth capability and potential trophic interactions with other microbial species. There are multiple approaches that can be used in concert to improve the prediction of growth requirements *a priori* and thus aim towards genome-specific media composition for gap filling in high-throughput GEM reconstruction. Computational tools that predict potential auxotrophies [40,41] can provide an initial starting point to constrain the media composition for gap filling. In this case, biomass precursors for which an auxotrophy has been identified can be added to the media prior to gap filling such that only transport reactions are added. By contrast, precursors that are known to be synthesized by the species can be removed such that the whole-synthesis pathway is gap filled. However, this approach depends on our knowledge of pathways and gene–protein–reaction annotations and is therefore limited by our incomplete knowledge as we are still uncovering new metabolic pathways and enzymes involved in biosynthesis of amino acids. For example, a recent study uncovered nine novel enzymes associated with amino acid biosynthesis by reconciliating experimental data with computational predictions across 10 different species [42]. Similarly, targeted gap filling has improved the prediction of known auxotrophies by reconciling experimental data with computational prediction in the gut microbiome [43]. A complementary and culture independent approach is to screen the exo-metabolome and profile accessible metabolites [44]. The most common techniques involve gas chromatography–mass spectroscopy, liquid chromatography–mass spectroscopy or nuclear magnetic resonance for targeted (i.e. profiling for specific metabolites) or untargeted (obtaining a whole metabolite footprint) metabolomics [44–46]. This approach has been used successfully to determine the available metabolites for nutrient uptake in flux balance simulations [47,48] and can be used to further constrain the available nutrient and biomass precursors in the media composition for automated gap filling.

High-throughput reconstruction of metabolic networks from MAGs is a promising and culture independent avenue to predict trophic interactions in complex microbial communities. Our analysis has shown that the appropriate selection of the media composition for gap filling during metabolic network reconstruction has profound consequences for the predicted auxotrophies. Using a minimal media formulation for gap filling generates prototrophic phenotypes as biosynthesis pathways are readily reconstructed whereas rich media formulations for gap filling promote auxotrophic phenotypes through the addition of transport reactions but omitting biosynthesis pathway reconstruction. Interestingly, the completeness of the MAGS does not influence the predicted auxotrophies of the reconstructed metabolic network in our cohort (electronic supplementary material, table S1). Nevertheless, the fraction of gap-filled reactions decreased with an increase in MAG completeness and when considering the substantial bias that gap filling algorithms may introduce [49], high-quality MAGs will result in more reliable metabolic network reconstructions.

In addition to KBase, there are numerous pipelines for semi-automated metabolic network reconstruction that employ diverse approaches, each having their own benefits and weaknesses [50]. Most of these tools use a bottom-up approach for reconstruction and gap filling [50] where missing reactions are added sequentially to ensure production of all biomass precursors. By contrast, CarveMe [23] uses a top-down approach based on a universal metabolic network template and retains reactions based on genetic evidence. A benefit of this approach is its media agnostic nature, resulting in GEMs that are not biased by the choice of media composition during reconstruction and provides an advantage for the reconstruction of uncultivated species for which experimental validation data cannot be obtained. CarveMe is incorporated in metaGEM [27], a pipeline that directly translates metagenomes via MAGs to functioning GEMs. Although this approach circumvents the issues caused by media composition during reconstruction, the authors note that metagenomes from complex communities (such as plant-associated, marine or soil communities) typically result in lower quality MAGs and thus pose a challenge for the top-down reconstruction approach. In addition, metabolic networks reconstructed with CarveMe may exhibit fewer auxotrophies due to the use of a manually curated universal template that lacks dead-end metabolites [50]. To increase the reliability of reconstructed metabolic networks from MAGs for community interaction studies, we encourage the use of computational tools *a priori* such as AuxoFind [40] or GapMind [41] to determine potential auxotrophies and combine this with exo-metabolomic data from the same sample used for obtaining the metagenome that forms the basis of the MAGs.

Trophic interactions have long been identified as crucial elements for microbial community assembly and stability [51]. Gaining mechanistic insights into microbial interaction often calls for a reductionist approach [52] where a few well-known species are combined in a laboratory setting under controlled conditions (e.g. synthetic ecology approaches). However, the inherent complexity of most natural microbial communities poses a challenge when using this approach and they are thus often studied using a holistic approach (e.g. meta-omic studies of microbial communities). Although interactions between different species in meta-omic studies can be inferred from co-occurrence analysis or correlations [53], these approaches do not permit mechanistic insights into the nature of the multi-species interactions. Translating metagenomes into functional GEMs via MAGs to simulate microbial communities is a promising avenue to bridge this holistic–reductionist divide and permit mechanistic insights into the functioning and resilience of microbial ecosystems. In addition to inferring interaction patterns, species-resolved metabolic community models provide novel avenues for targeted isolation of so far uncultured species or guide the search for new metabolic interventions when fighting clinically relevant pathogens. A recent study used a GEM of *Klebsiella pneumoniae* to reveal its increased reliance on l-valine uptake [54]. However, extending this method to community metabolic models based on MAGs necessitates confidence in the whole reconstruction pipeline, especially concerning potential cross-feeding metabolites and prediction of auxotrophies that lie at the heart of microbial community interactions.

## 3. Methods

### 3.1. Isolate genomes and metagenome-assembled genomes

We selected eight bacterial isolates; four from the Firmicutes phylum and four from the Gammaproteobacteria class and downloaded their genomes from the public KBase [34] genome database. The MAGs used in this study were assembled in a previous study [55] and are available in NCBI BioProject database (BioProject accession number PRJNA850115). In short, all MAGs in this study were assembled from urban metagenomes found in the TerrestrialMetagenomeDB (https://webapp.ufz.de/tmdb/ [56]) using the Multi-Domain Genome Recovery (MuDoGeR) [57]. Two MAGs were selected based on their GTDB-tk (v2) [58] predicted taxonomy for comparison with the respective isolate genomes (*E. coli* K12 MG1655 and *P. putida* B6-2), and five MAGs were selected randomly from the set of Gammaproteobacteria and Firmicutes MAGs in BioProject PRJNA850115. MAGs in the previously mentioned study by Magnúsdóttir *et al.* were clustered into genomic OTUs (gOTUs) using the MuDoGeR tool, which uses FastANI [59] to cluster MAGs into gOTUs based on average nucleotide identity of 95. We selected one of the larger gOTUs representing the species *Pseudomonas nitrititolerans* and created GEMs from all 106 MAGs. This gOTU species was selected based on the number of MAGs that it contained and the large range of genome completeness (determined using CheckM [60]).

### 3.2. Reconstruction and analysis of genome-scale metabolic models

We reconstructed all genome-scale metabolic models in KBase [34]. We imported the Genbank genome fasta files into KBase and annotated all genomes using the ‘Annotate Multiple Microbial Genomes with RASTtk - v1.073 (v1.9.5)’ app [35]. We subsequently reconstructed all metabolic networks using the ‘Build Multiple Metabolic Models (v2.0.0)’ app with four different media compositions for gap filling. These include two defined minimal media (C-Citrate and C-D-Glucose), one defined rich medium (ArgonneNMSMedia [61]) and one rich complex medium (ArgonneLBMedia). Finally, we used the ‘Model Comparison (v1.0.0)’ app to obtain pathway-resolved gap-filled reactions. We subsequently imported all models (SBML) and media formulations (CSV) into Matlab R2022a and screened for missing biomass precursors on all different media compositions using the ‘biomassPrecursorCheck’ from the COBRA toolbox v3.0 [62] to map and calculate the total number of predicted amino acid auxotrophies.

## Data Availability

Reconstructed genome-scale metabolic models and media compositions used in this manuscript are available from https://doi.org/10.5281/zenodo.7515886 [63]. The data are provided in the electronic supplementary material [64].
